# Metal interactions of α-synuclein probed by NMR amide-proton exchange

**DOI:** 10.3389/fchem.2023.1167766

**Published:** 2023-05-02

**Authors:** Mario Gonzalez-Garcia, Giuliana Fusco, Alfonso De Simone

**Affiliations:** ^1^ Department of Life Sciences, Imperial College London, London, United Kingdom; ^2^ Centre for Misfolding Diseases, Department of Chemistry, University of Cambridge, Cambridge, United Kingdom; ^3^ Department of Pharmacy, University of Naples “Federico II”, Naples, Italy

**Keywords:** α-synuclein, NMR, amide exchange, aggregation, metal interaction

## Abstract

The aberrant aggregation of α-synuclein (αS), a disordered protein primarily expressed in neuronal cells, is strongly associated with the underlying mechanisms of Parkinson’s disease. It is now established that αS has a weak affinity for metal ions and that these interactions alter its conformational properties by generally promoting self-assembly into amyloids. Here, we characterised the nature of the conformational changes associated with metal binding by αS using nuclear magnetic resonance (NMR) to measure the exchange of the backbone amide protons at a residue specific resolution. We complemented these experiments with ^15^N relaxation and chemical shift perturbations to obtain a comprehensive map of the interaction between αS and divalent (Ca^2+^, Cu^2+^, Mn^2+^, and Zn^2+^) and monovalent (Cu^+^) metal ions. The data identified specific effects that the individual cations exert on the conformational properties of αS. In particular, binding to calcium and zinc generated a reduction of the protection factors in the C-terminal region of the protein, whereas both Cu(II) and Cu(I) did not alter the amide proton exchange along the αS sequence. Changes in the R_2_/R_1_ ratios from ^15^N relaxation experiments were, however, detected as a result of the interaction between αS and Cu^+^ or Zn^2+^, indicating that binding to these metals induces conformational perturbations in distinctive regions of the protein. Collectively our data suggest that multiple mechanisms of enhanced αS aggregation are associated with the binding of the analysed metals.

## 1 Introduction

α-synuclein (αS) is an intrinsically disordered protein (IDP) that is primarily expressed in neuronal cells and whose aggregation is strongly associated with debilitating neurodegenerative diseases collectively known as synucleinopathies, which include Parkinson’s disease (PD), dementia with Lewy bodies and multiple system amyotrophy (Spillantini et al., 1997; Uversky and Eliezer, 2009; Luk et al., 2012; Lashuel et al., 2013; Chiti and Dobson, 2017; Fusco et al., 2017; Newberry et al., 2020). Amyloid fibrils of αS are indeed the major constituents of aberrant inclusions, designated as Lewy bodies, forming in neurons of patients affected by PD (Lashuel et al., 2013; Lee and Masliah, 2015). There are also genetic links between synucleinopathies and αS, with a number of missense mutations, duplications and triplications of the gene encoding αS being identified in association with familial forms of early onset PD (Singleton et al., 2003; Chartier-Harlin et al., 2004; Roberts and Brown, 2015).

In its physiological form, αS is predominantly localized at the presynaptic terminals of neurons, where it has been associated with the regulation of the homeostasis of synaptic vesicles (Auluck et al., 2010; Fusco et al., 2018), however, its exact function remains debated. It is generally believed that the biological activity of αS is inextricably linked to its ability to bind biological membranes (Snead and Eliezer, 2014). This interaction is recursively involved in the major putative neuronal roles of αS (Burre, 2015), and has been shown to promote αS aggregation (Auluck et al., 2010) and the mechanisms of neurotoxicity induced by its aggregates (Fusco et al., 2017).

A fundamental interaction of αS involves metal ions, including divalent cations such as Cu^2+^, Mn^2+^, Zn^2+^, as well as Ca^2+^. The latter divalent cation, a key messenger in neurotransmission, was also shown to alter the conformations and membrane interactions of αS (Lautenschlager et al., 2018). There is a crucial interest on the role of metals in PD as their dishomeostasis is increasingly recognised to play a critical role in the development of this disease. In addition, numerous evidences indicate that metal interactions promote aberrant aggregation of αS (Fink, 2006; Binolfi et al., 2008; Binolfi et al., 2010; Deas et al., 2016), including Ca^2+^ (Stephens et al., 2020), Mn^2+^ (Uversky et al., 2001; Verina et al., 2013), Zn^2+^ (Sato et al., 2013) and Cu^2+^ (Montes et al., 2014). αS has also been shown to interact with Cu^+^, a binding implicated in the formation of reactive oxygen species inducing toxicity in dopaminergic neurons (Wang et al., 2010). The modes of metal binding by αS are variegated. Generally, the significant presence of negatively charged residues in the C-terminal region of αS promotes electrostatic interactions with cations, whereas His 50 and Met residues in the N-terminal region provide additional interaction loci for some metal cations (Supplementary Figure S1).

In order to understand the role of metal binding in the pathophysiology of αS it is therefore critical to characterise the subtle conformational alterations of αS associated with these interactions. It is indeed currently generally acknowledged that long-range interactions between the negatively charged C-terminus and the positively charged N-terminal region of αS promote an aggregation-resistant conformational ensemble whereby the amyloidogenic NAC region is partially protected from engaging in dangerous self-assembly and aggregation (Dedmon et al., 2005). Alterations of this conformational ensemble, such as, for example, those induced by Ca^2+^ binding at the C-terminal region, have been shown to modify the properties of αS in such a way to increase the exposure of the NAC, ultimately leading to its aggregation (Stephens et al., 2020).

The metal interaction by αS has been extensively studied using nuclear magnetic resonance (NMR) (Rasia et al., 2005; Binolfi et al., 2006; Binolfi et al., 2008; Binolfi et al., 2010; Binolfi et al., 2011; Miotto et al., 2014; Miotto et al., 2015; Villar-Pique et al., 2017; Lautenschlager et al., 2018; Gonzalez et al., 2019; Pontoriero et al., 2020), and here we applied NMR to probe the backbone protection factors of αS upon interaction with divalent (Ca^2+^, Cu^2+^, Mn^2+^, and Zn^2+^) and monovalent (Cu^+^) metal ions. In particular, we used phase-modulated CLEAN chemical exchange (CLEANEX) to directly monitor the H-H exchange of amide protons with the solvent, as previously employed for the isolated αS (Okazaki et al., 2013), and complemented these experiments with ^15^N relaxation to collectively probe conformational dynamics on various of timescales. The data collectively mapped the effects that individual metal ions exert on the conformational properties of αS upon binding, thereby suggesting the nature of the structural perturbations by which these metals trigger αS aggregation.

## 2 Materials and methods

### 2.1 αS expression and purification

αS was expressed in BL21 *Escherichia coli* using plasmid pT7-7 and purified as previously described following an established protocol (Fusco et al., 2016; Fusco et al., 2017). N-terminal acetylation of αS was obtained by co-expression with a plasmid encoding the components of the NatB complex (Maltsev et al., 2012). ^15^N and/or ^13^C-labelled αS was expressed in M9 minimal media containing 1 g/L of ^15^N ammonium chloride and 3 g/L of ^13^C-glucose. To enhance the N-terminal acetylation of αS, 1 g of ISOGRO^®^^15^N-^13^C was added. The bacterial culture was supplemented with 100 µg/mL ampicillin, together with 100 µg/mL chloramphenicol for cultures coexpressing both plasmids where N-terminal acetylation was desired, and incubated at 37°C under constant shaking at 200 rpm to an OD600 of 0.6–1.0. Expression was induced through the addition of 1 mM isopropyl β-D-1-thiogalactopyranoside (IPTG) and overnight incubation under constant shaking at 28°C.

The cells were then harvested by centrifugation at 6,200 x g for 20 min at 4°C (Beckman Coulter Brea, United States), the cell pellets were resuspended in 1 M PBS and centrifuged again at 24 500 x g for 1 h at 4°C. Each pellet was then resuspended in lysis buffer (10 mM Tris-HCl pH 7.7, 1 mM EDTA and ½ of an EDTA-free complete™ protease inhibitor cocktail tablet) and lysed by sonication on ice (2 s on, 4 s off, total time 8 min). The sonicated samples where then centrifuged at 24 500 x g for 30 min at 4°C to remove the cell debris as pellets. The supernatant was then heated for 20 min at 94°C to precipitate heat-sensitive proteins. A further centrifugation step at 24 500 x g for 30 min at 4°C followed to remove the precipitated protein fraction. Subsequently, the supernatant was treated with 10 mg/mL of streptomycin sulfate to induce DNA precipitation. The solution was stirred for 15 min at 4°C and centrifuged again at 24 500 x g for 30 min at 4°C. In order to precipitate and recover αS, ammonium sulfate was slowly added to a concentration of 361 mg/mL and stirred for 30 min at 4°C. A final centrifugation step at 24 500 x g for 30 min at 4°C recovered the precipitated protein, which was then resuspended in 25 mM Tris-HCl, pH 7.7 and dialysed in the same buffer overnight at 4°C.

The dialysed solution was then loaded onto an anion exchange column (Q Sepharose HP HiScale 26/20, 6–7 cm, Cytiva) and eluted with a 0–1.5 M NaCl step gradient. The eluted fractions containing αS were concentrated using Vivaspin filter concentrators (Sartorius Stedim Biotech, Göttingen, Germany) and filtered through a 0.22 μm filter to remove any precipitates. The protein was then further purified by loading onto a size exclusion column (HiLoad 16/60 Superdex 75 pg, GE Healthcare, Little Chalfont, United Kingdom). The pooled fractions were concentrated and dialysed in 25 mM Tris-HCl, pH 7.0. Stored αS samples that had previously been dissolved in buffers containing metal ions were dialysed three times to remove any traces of those metals. The purity of the fractions was monitored after every major purification step by SDS-PAGE and the concentration of monomeric αS determined by the absorbance at 280 nm using a molar extinction coefficient of 5960 M^−1^ cm^−1^ with a Nanodrop.

### 2.2 NMR setup

All NMR measurements in this study were carried out using a Bruker 800 MHz spectrometer equipped with a triple resonance HCN cryo-probe (Bruker, Coventry, United Kingdom). Residue assignment of NMR resonances was obtained from previous studies (Fusco et al., 2016; Fusco et al., 2017). ^1^H-^15^N spectra, including HSQC and CLEANEX, were performed using a data matrix consisting of 2048 (t2, ^1^H) × 220 (t1, ^15^N) complex points and 64 scans. NMR spectra were acquired using Topspin 3.6.0 (Bruker, AXS GmBH, DE) and processed with CCPNmr v2.0. In order to assess if during the NMR measurements the monomer concentration is reduced as a result of protein aggregation, ^1^H-^15^N-HSQC spectra were measured at the start and end of the dataset collection, showing no significant changes in the peak intensities and frequencies (Supplementary Figure S2). All the NMR spectra were recorded at 10°C using 25 mM Tris-HCl at pH 7.0.

### 2.3 Phase-modulated CLEAN chemical exchange NMR experiments

CLEANEX probes the chemical exchange between fast exchangeable hydrogen atoms with water (Hwang et al., 1998). This phenomenon can directly probe the solvent accessibility of specific groups of proteins (De Simone et al., 2011; Fusco et al., 2022). Measurements were recorded at 283K, a condition that allows for excellent signal to noise for the ^1^H-^15^N resonances in HSQC spectra of αS. CLEANEX experiments were measured using N-terminally acetylated or non-acetylated αS (415 μM) incubated with the different monovalent and divalent cations considered in this study. This NMR technique allows an estimation of the exchange rates from the slope of the linear interpolation of the intensities of amide peaks from spectra recorded at different mixing times τ_m_ (5, 10, 15, 20, and 25 ms). In particular, the volumes V_i_ of the peaks were normalized relative to those of the corresponding ^1^H-^15^N-HSQC peaks V_0_. By plotting V_i_/V_0_ as a function of τ_m_, *k*_*ex*_ can be defined from the slopes of the linear interpolation. The *k*_*ex*_ values were normalised with theoretical values calculated from the sequence (Connelly et al., 1993) and assuming that the peptide chain is in a random coil conformation (*k*_*int*_) to obtain the protection factor logP from the logarithm of *k*_*int*_/*k*_*ex*_. This data analysis is formally applied under the EX2 regime of amide exchange, which was previously demonstrated in CLEANEX experiments of isolated αS at pH 7.0 (Okazaki et al., 2013). Calculated error bars in our data analysis represent the fitting error in the calculation of the *k*_*ex*_ for each residue.

### 2.4 Chemical shift perturbations (CSP) in ^1^H-^15^N-HSQC

^1^H-^15^N-HSQC spectra were measured at 283K using αS samples dissolved in 25 mM Tris-HCl, pH 7.70 and/or in combination with the monovalent and divalent cations discussed in this study. Mean weighted chemical shift (MWCS) profiles were calculated as √[(Δδ^1^H)^2^ + (Δδ^15^N/10)^2^]. For the intensity (I/I_0_) and MWCS analyses, only well-resolved and unambiguously assigned HSQC peaks were utilised. Data were processed and analysed using the CCPNmr Analysis software. Resonance assignments were done as with CLEANEX measurements.

### 2.5 Transverse and longitudinal relaxation NMR experiments

Transverse (T2) and longitudinal (T1) ^15^N relaxation experiments were acquired using standard pulse sequences (Farrow et al., 1994), including the watergate sequence (Piotto et al., 1992) to enhance water suppression. R_1_ and R_2_ values were obtained by fitting the data to an exponential decay function with single relaxation delays (τ delays: 4, 30, 70, 120, 200, 300, 400, 500, 700, 1,000 ms; τ delays: 0, 20, 40, 50, 60, 80, 100, 120, 140, 160, 170, and 200 ms). Experiments were recorded as data matrices consisting of 2048 (t_2_, ^1^H) × 220 (t_1_, ^15^N) complex points. Relaxation was measured at 283K on samples of N-terminally acetylated αS (400 μM) incubated with monovalent and divalent cations considered in this research, using a Bruker spectrometer operating at a ^1^H frequency of 800 MHz equipped with a triple resonance HCN cryo-probe (Bruker, Coventry, United Kingdom). Resonance assignments were done as with CLEANEX measurements. Calculated error bars represent the fitting error in the calculation of the *Kex* for each residue.

### 2.6 Experimental procedure to obtain Cu^+^

Copper was reduced by pre-incubation using an excess of 10 mM sodium ascorbate. Considering the concentration of 85 μM of copper used in this study, the molar ratio of copper:ascorbate was set to 1:120. The reduced copper solution, mixed with sodium ascorbate, was then added to the αS sample. A thin layer of mineral oil was added on top of the sample to prevent changes in the resonances of methionine residues arising from air oxidation effects.

## 3 Results

In order to investigate the subtle perturbations that metal ions exert on the conformational properties of αS, we employed biomolecular NMR to elucidate the nature of the weak binding with Ca^2+^, Zn^2+^, Cu^+^, Cu^2+^, and Mn^2+^. Our approach was based on a comprehensive analysis of the metal interactions by αS, including the map of the transient protein-metal contacts, through chemical shift perturbations (CSP) in the ^1^H-^15^N-HSQC spectra, and the effects of the binding on slow (millisecond timescale) and fast (nanosecond timescale) protein dynamics, respectively probed using CLEANEX-PM and ^15^N relaxation spectra. The results indicate that the modes of binding between these metals and αS can be markedly different, including the protein regions involved in the interactions and the consequent perturbations in the conformational ensemble of αS.

### 3.1 Calcium interaction

**FIGURE 1:**
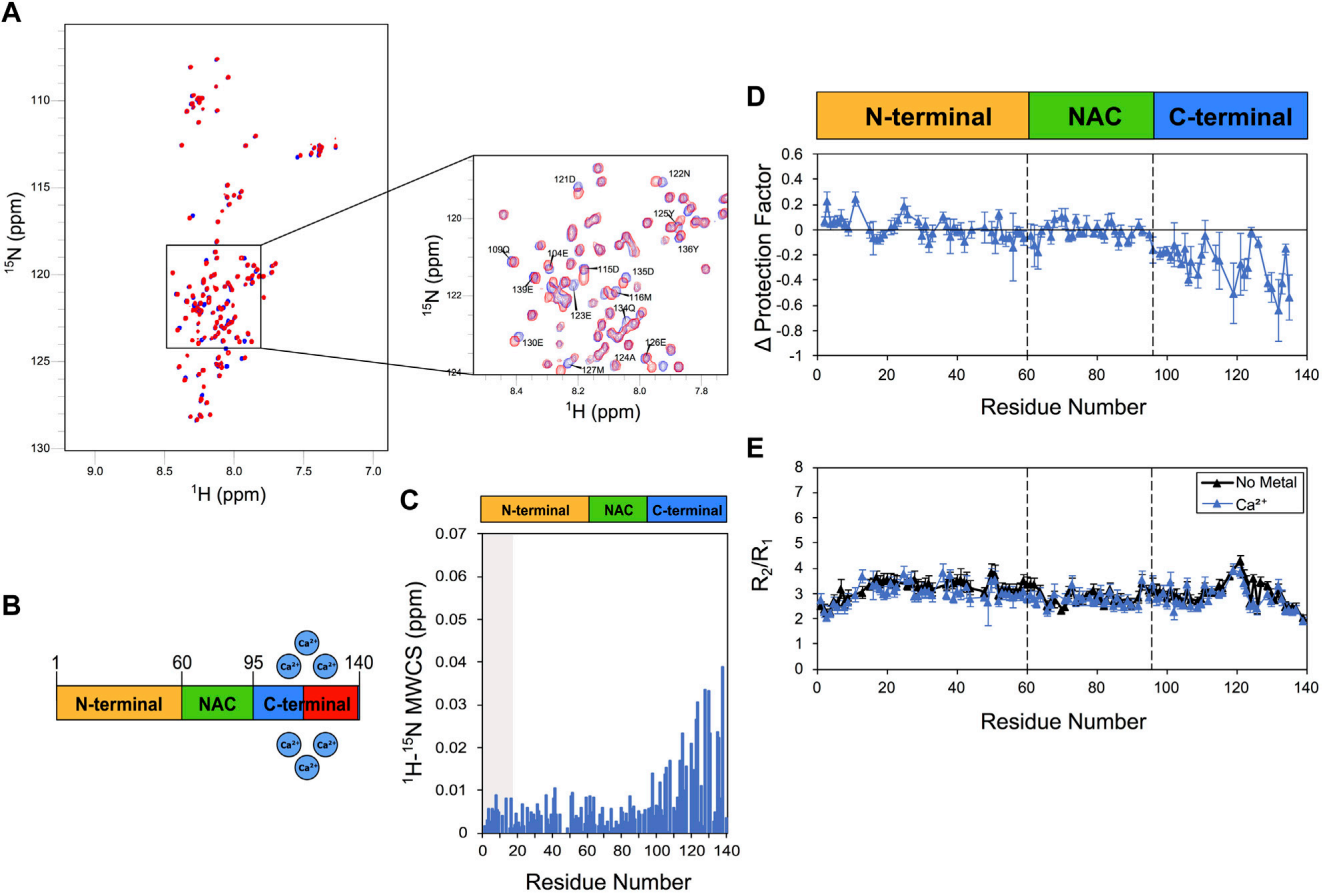
NMR analysis of Ca^2+^ binding to αS. Experiments were performed at 10°C in 25 mM Tris buffer and a pH of 7.0, and using concentrations of αS and Ca^2+^ of 415 μM and 10 mM, respectively. **(A)** ^1^H-^15^N HSQC spectra of αS in the presence (blue) and absence (red) of calcium. **(B)** Schematic depicting Ca^2+^ binding by αS. Red regions in the scheme indicate major CSP along the αS sequence upon metal binding. **(C)** Mean weighted CSP (^1^H-^15^N MWCS) of αS as a result of the calcium binding. The grey box denotes the first 15 residues of the protein. **(D)** Alteration in the LogP values of αS as a result of Ca^2+^ binding as measured in CLEANEX experiments (Supplementary Figures S5, S6). These values are calculated for each αS residue as the logP of the metal bound state minus the logP of the isolated protein state. **(E)** R_2_/R_1_ values from ^15^N relaxation data of αS in the presence (blue) and absence (black) of calcium (raw data in Supplementary Figure S7). Error bars are calculated from the fitting errors in R_1_ and R_2_ measurements. Dotted lines delineate the different regions (N-terminal, NAC and C-terminal) along the sequence of αS.

We first employed the combination of CSP, CLEANEX-PM and ^15^N relaxation to analyse the calcium binding by αS (Figure 1). In agreement with previous studies (Lautenschlager et al., 2018; Stephens et al., 2020), Ca^2+^ was found to induce CSP in the acidic C-terminus of αS under the present experimental conditions (Figure 1). This binding is mediated by Asp and Glu residues that are abundant in the region 110–140 (Supplementary Figure S1). In order to sample slow protein motions, we measured amide exchange protection factors through CLEANEX NMR. These experiments revealed high LogP values in correspondence of the C-terminal region of the isolated αS (Supplementary Figure S3), an observation that is in line with previous NMR studies (Okazaki et al., 2013) as well as orthogonal measurements of mass spec (Stephens et al., 2020). This pattern of C-terminal protection, which is conserved in both N-terminally acetylated and non-acetylated forms of αS (Supplementary Figure S3), has been ascribed to the local concentration of negative charges in the αS sequence (Okazaki et al., 2013). In the presence of calcium, CLEANEX revealed alterations of the protection factors of αS, primarily in correspondence of the C-terminal region of the protein where reductions up to 0.6 in LogP values were observed. These data are in apparent contrast with previous mass spec analyses (Stephens et al., 2020; Seetaloo et al., 2022), likely due to differences in the timescales of the exchange process probed by the two techniques. Both experiments, however, provide converging indication that calcium binding disrupts the electrostatically driven transient interactions between the N-terminal and C-terminal regions of αS, which were observed using NMR paramagnetic relaxation enhancement (Dedmon et al., 2005).

To further study the Ca^2+^ interaction by αS, we then employed ^15^N-relaxation. The data showed no significant alterations in the R_2_/R_1_ values upon calcium binding, including residues of the C-terminal region (Figure 1E), suggesting that the metal interaction induces no specific conformational exchange in the intermediate NMR timescale. We noted a slight increase in the longitudinal relaxation rates (R_1_) in correspondence of the C-terminal region of αS (residues 105–140), which is consistent with the region showing the strongest CSP and LogP reductions upon calcium interaction. Taken together these data indicate that calcium binding perturbs the conformational ensemble of αS by reducing LogP values in the C-terminal region, a result that is compatible with a destabilization of the transient interaction between N- and C- terminal regions of αS.

### 3.2 Copper binding

**FIGURE 2:**
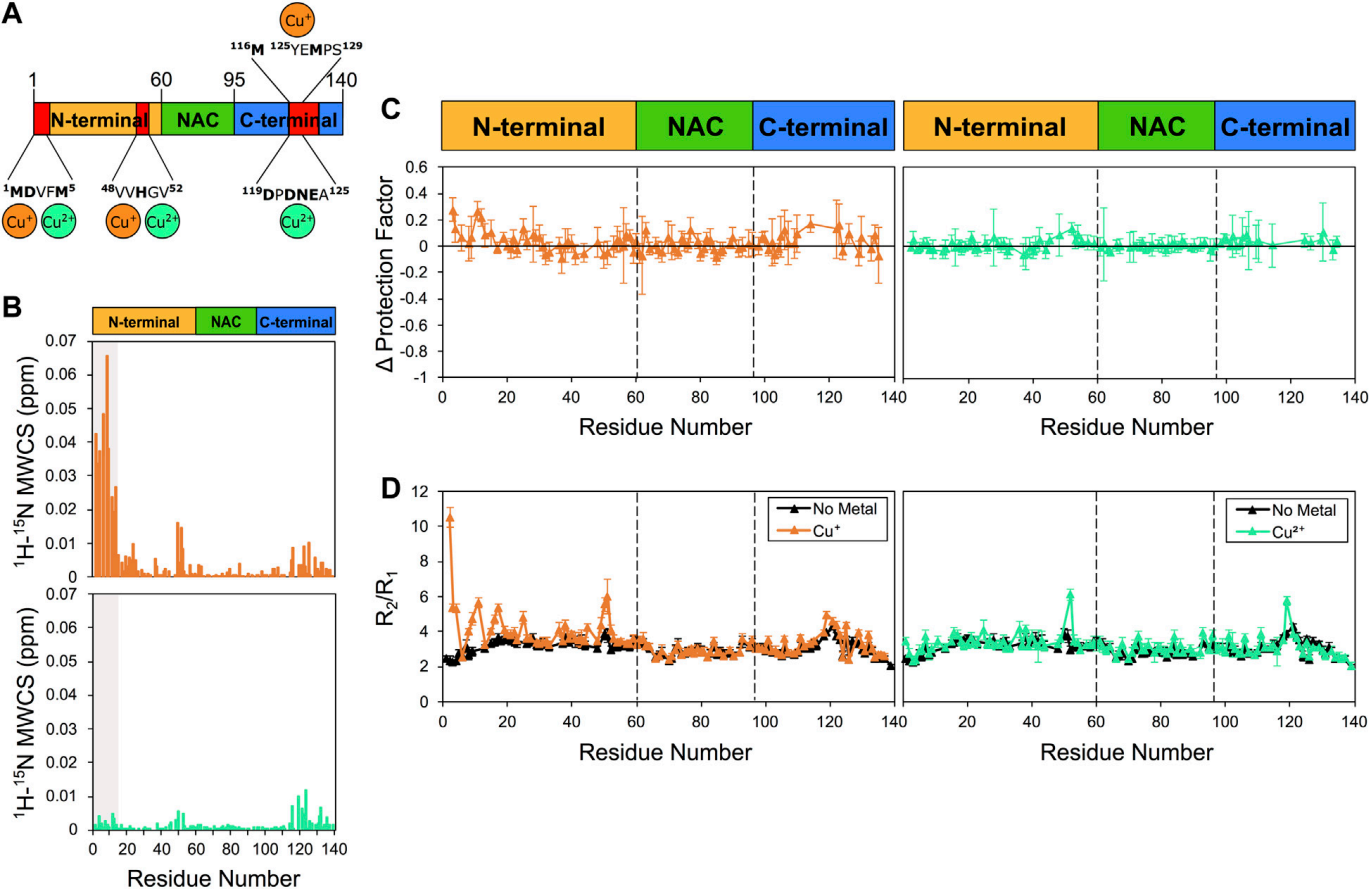
NMR analysis of copper binding to αS. Experiments were performed at 10°C in 25 mM Tris buffer and a pH of 7.0, and using concentrations of αS and Cu^+^ (or Cu^2+^) of 415 μM and 85 μM, respectively. **(A)** Schematic depicting Cu^+^ and Cu^2+^ binding by αS. Red regions in the scheme indicate major CSP along the αS sequence upon metal binding. **(B)** Mean weighted CSP (^1^H-^15^N MWCS) of αS as a result of the copper binding. Top and bottom panels for Cu^+^ and Cu^2+^ binding, respectively. The grey boxes denote the first 15 residues of the protein. **(C)** Alteration in the LogP values of αS as a result of copper binding. Left and right panels for Cu^+^ and Cu^2+^ binding, respectively. These values are calculated for each αS residue as the logP of the metal bound state minus the logP of the isolated protein state. **(D)** R_2_/R_1_ values from ^15^N relaxation data of αS in the presence of Cu^+^ (orange, left panel), Cu^2+^ (green, right panel) and in the isolated protein (black) (raw data in Supplementary Figure S7). Error bars are calculated from the fitting errors in R_1_ and R_2_ measurements. Dotted lines delineate the different regions (N-terminal, NAC, and C-terminal) along the sequence of αS.

We then studied the αS/copper binding, a relevant interaction in the context of synucleionpathies (Rasia et al., 2005; Sung et al., 2006; Miotto et al., 2015). For these experiments, in order to reduce broadening of the NMR resonances due to paramagnetic effects, we employed a 1:5 ratio of copper:αS (85 μM: 415 μM), and maintained this ratio for both Cu^+^ and Cu^2+^ analyses. In the case of Cu^+^, in order to ensure the optimal oxidation state of copper, we used an excess of sodium ascorbate (see Section 2). The latter was found to induce no conformational changes in αS, as the ^1^H-^15^N HSQC of the protein resulted unperturbed in the presence of the reducing agent. By contrast, the presence of Cu^+^ and Cu^2+^ was found to induce considerable CSP to the ^1^H-^15^N HSQC spectrum of αS, particularly in three regions of the protein sequence that include the N-terminus, the residues flanking His50, and the C-terminus (Figure 2). The strongest effects were observed in the case of Cu^+^, and particularly in correspondence of the N-terminal 13 residues of αS. Despite the considerable levels of CSP, no significant alterations of the protection factors of αS were detected upon copper interaction (Figure 2C). More specifically, only a slight increase in the protection factors at the N-terminus of αS upon Cu^+^ binding was observed up to a value of +0.3 in LogP, whereas binding of Cu^2+^ did not induce any significant change in the measured protection factors of the protein. While monomer depletion was not observed during the present measurements (Supplementary Figure S2), indicating that no significant aggregation occurred during the data acquisition, it is possible that dimerization events induced by Cu^+^ (Abeyawardhane et al., 2018) may have contributed to the changes in the measured protection factors.

When analysing the ^15^N relaxation of αS upon the interaction with copper, we observed a strong increase in R_2_/R_1_ values in some protein regions in the presence of Cu^+^ (Figure 2D). These changes in R_2_/R_1_ values, which resulted particularly evident in correspondence of the N-terminal region and in proximity of His50, indicate that the interaction with Cu^+^ induces conformational exchange in the intermediate NMR timescale. We also noted that Cu^+^ induces a mild reduction in R_1_ values, which is significant primarily in the N-terminal region of αS, whereas binding of Cu^2+^ did not induce significant changes in R_2_/R_1_ values, except in proximity of His50 and Asp121.

### 3.3 Zinc and manganese interaction

**FIGURE 3:**
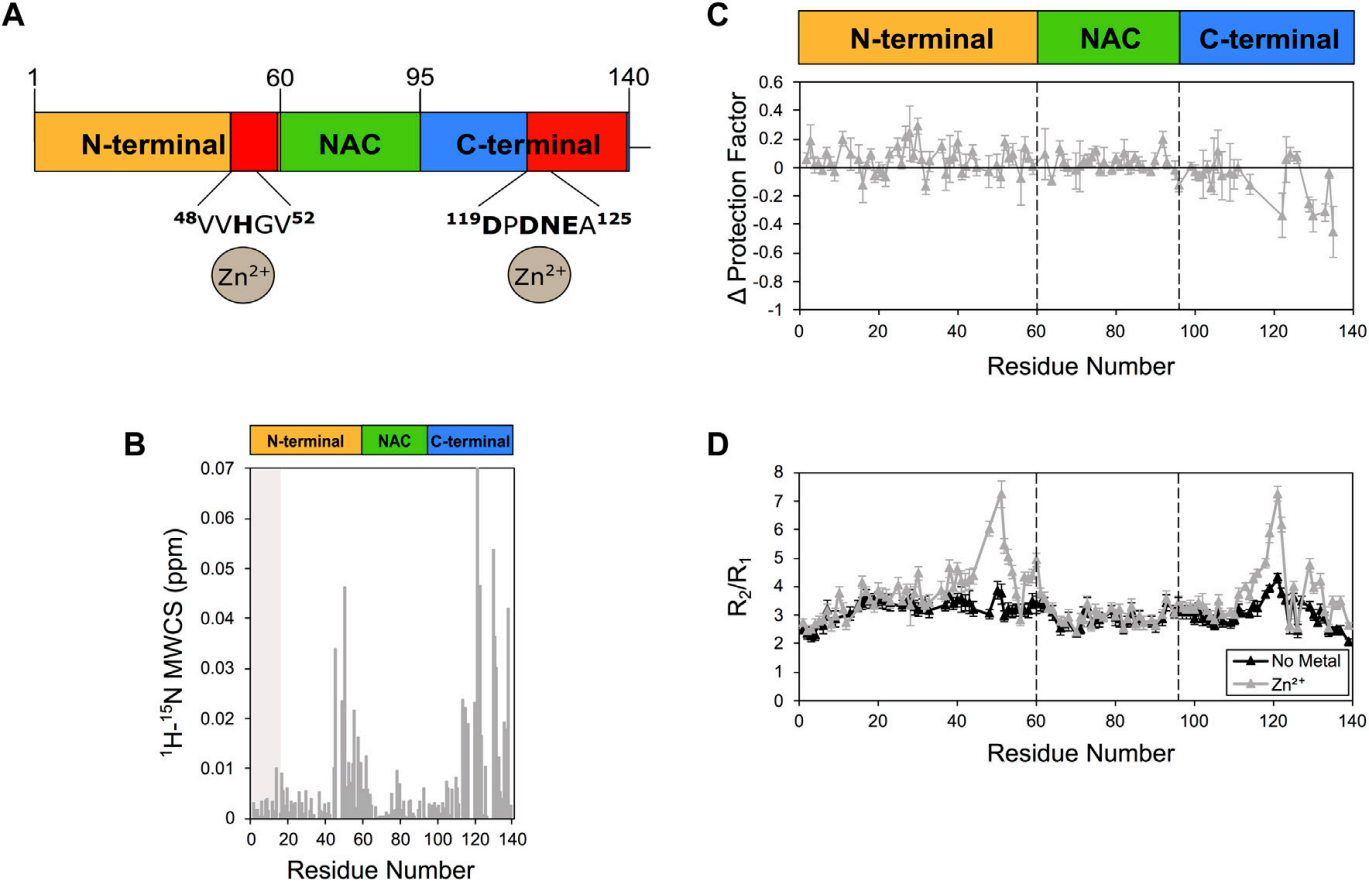
NMR analysis of Zn^2+^ binding to αS. **(A)** Schematic depicting Zn^2+^ binding by αS. Red regions in the scheme indicate major CSP along the αS sequence upon metal binding. **(B)** Mean weighted CSP (^1^H-^15^N MWCS) of αS as a result of the zinc binding. The grey box denotes the first 15 residues of the protein. **(C)** Alteration in the LogP values of αS as a result of Zn^2+^ binding. These values are calculated for each αS residue as the logP of the metal bound state minus the logP of the isolated protein state. **(D)** R_2_/R1 values from ^15^N relaxation data of αS in the presence (gray) and absence (black) of zinc (raw data in Supplementary Figure S8). Error bars are calculated from the fitting errors in R_1_ and R_2_ measurements. Dotted lines delineate the different regions (N-terminal, NAC, and C-terminal) along the sequence of αS. Experiments were performed at 10°C in 25 mM Tris buffer and a pH of 7.0, and using concentrations of αS and Zn^2+^ of 415 μM and 2.1 mM, respectively.

When we probed the interaction between αS and Zn^2+^. The experiments indicated strong perturbations of the ^1^H-^15^N-HSQC spectrum of αS (Figure 3), with major CSP found in proximity of His50 and Asp121 (Figure 3B). Zinc interaction was found also to induce alterations of the protection factors of αS, with significant reductions of the LogP values of the C-terminal region of the protein (Figure 3C). In addition, Zn^2+^ binding strongly perturbed the relaxation properties of αS, with significant alterations in R_2_/R_1_ values in proximity of His 50 and Asp121 (Figure 3D). Collectively these NMR data indicate specific zinc binding in two regions of αS resulting in conformational exchange in the intermediate timescale in the regions flanking residues 50 and 121 as well as enhanced solvent exchange in the C-terminal region of the protein.

Finally, the incubation of Mn^2+^ generated very minor CSP primarily localised in the C-terminal region of αS (Supplementary Figure S4). Because of broadening effects upon manganese binding, in the C-terminal region of αS protection factors could be obtained only for very few residues (Supplementary Figure S4C), indicating generally no perturbation in the local solvent exchange. By contrast, enhanced R_2_/R_1_ values were observed in correspondence in the N-terminal and C-terminal regions of the protein (residues M1, G25, A27, G36, G51, Q99, L100, G101, K102, N103, E104, E105, A107, E110, G111, and I112). It is worth noting that Mn^2+^ can induce enhanced transverse relaxation in NMR resonances, thereby possibly altering R_2_/R_1_ values as a result of the paramagnetic effect. In this case, changes in R_2_/R_1_ may not exclusively reflect alterations in the conformational ensemble of αS.

## 4 Discussion

A number of evidences exist about the role of metal ions in the underlying mechanisms at the onset and development of PD (Carboni and Lingor, 2015; Bjorklund et al., 2018; Vellingiri et al., 2022). Alterations in copper homeostasis in neuronal cells, for example, have been associated with processes of neurodegeneration, including oxidative stress, dopamine oxidation, mitochondrial impairment (Bisaglia and Bubacco, 2020). Long-term exposure to manganese, copper and other metals is also known to enhance the risk of developing PD (Caudle, 2017), and it is now clear that different metal ions can act synergistically in inducing pathogenic processes in PD (Bjorklund et al., 2018). Despite these evidences, however, the role of metal dishomeostasis in PD is not fully understood and remains strongly debated. It has been extensively shown that metals can enhance the aggregation of αS by inducing the misfolding of αS into amyloid-prone species promoting fibrillization (Binolfi et al., 2006; Fink, 2006; Binolfi et al., 2008; Binolfi et al., 2010; Deas et al., 2016). The enhancement of αS aggregation has been observed in conjunction with numerous cations such as Ca^2+^ (Stephens et al., 2020), Mn^2+^ (Uversky et al., 2001; Verina et al., 2013), Zn^2+^ (Sato et al., 2013), Cu^2+^ (Montes et al., 2014) and Cu^+^ (Wang et al., 2010). We here focussed on these specific metals to aim at a detailed characterisation of their binding modes with αS. In order to generate new understanding of the effects of these interactions on the conformational properties of αS, using NMR CLEANEX we probed how the metals affect the amide protection factors of the protein. These experiments are specifically tailored to probe H/H exchange in solvent exposed regions of protein molecules (De Simone et al., 2011; Fusco et al., 2022) and IDPs (Hwang et al., 1998; Okazaki et al., 2013). Other NMR measurements of proton exchange of αS have shown that the cellular environment does not alter the rates of exchange (Smith et al., 2015), making this technique a fine probe of the conformational properties of αS in the crowded cellular environment. Backbone amide exchange is indeed specifically sensitive to slow backbone dynamics in proteins and perturbations of this process provide evidence of key conformational changes in IDPs, such as, for example, the formation of local hydrogen bonds.

Our data indicate that the individual metal ions have distinctive modes of binding with αS and induce specific perturbations of its amide protection factors. The binding signatures of each metal are even more unique when considering CSP and ^15^N relaxation data in addition to LogP. In particular, strong effects on the protection factors were observed in the presence of Ca^2+^ and Zn^2+^, with both cases associated with a reduction of LogP values of the C-terminal region of αS. In the presence of calcium, CSP were observed primarily in the C-terminal region of αS, suggesting only a local involvement in the metal binding, whereas upon zinc interaction CSP were observed also in the region proximal to His50. Moreover, calcium binding did not induce significant perturbations in ^15^N relaxation of αS while zinc was found to strongly enhance R_2_/R_1_ values in proximity of residue His50 and the C-terminal region of αS. These data indicate that the conformational changes that zinc induces on αS are different from those induced by calcium, with the first affecting the local conformations in two spots of the protein and the second influencing primarily the properties of the C-terminal region. In addition to zinc and calcium binding, we also found that copper-αS interactions have unique signatures. Copper binding indeed generates CSPs in three regions of the protein, including the N-terminus, the region in proximity of residue His 50 and the C-terminus, with perturbations induced by Cu^+^ found to be significantly stronger than those associated with Cu^2+^. The incubation with both types of copper cations did not affect significantly the protection factors of αS, whereas conformational changes probed by ^15^N relaxation indicated rearrangements of the N-terminal region upon Cu^+^ as revealed by R_2_/R_1_ values.

Taken together our results indicate that, although all the metal ions here studied accelerate αS aggregation, they attain different binding modes with the protein suggesting that multiple mechanisms of enhanced aggregation may occur as a result of these interactions. Understanding the nature of these mechanisms is therefore critical if we are to reveal the connection between metal dis-homeostasis and αS aggregation in the context of PD. A key challenge in this research area will be the characterization of synergic effects of the metal ions in their multiple interactions with αS, and how these are related with the various phases of the normal and pathological neuronal activity.

## Data Availability

The original contributions presented in the study are included in the article/Supplementary Material, further inquiries can be directed to the corresponding authors.
