# Supplementary material for: Metal interactions of α-synuclein probed by NMR amide-proton exchange
Source: Front Chem. 2023 May 2;11:1167766. doi: 10.3389/fchem.2023.1167766 (PMC10187754; doi:10.3389/fchem.2023.1167766)
Supplement: Supplementary file 1 [file Presentation1.pdf]

Supplementary Materials

Metal interactions of α-synuclein probed by NMR amide-proton exchange

Mario Gonzalez-Garcia[1], Giuliana Fusco[2] and Alfonso De Simone[1,3]

[1] Department of Life Sciences, Imperial College London, South Kensington, SW7 2AZ London, UK
[2] Centre for Misfolding Diseases, Department of Chemistry, University of Cambridge, Lensfield Road, CB2 1EW Cambridge, UK
[3] Department of Pharmacy, University of Naples, Via Montesano 49, 80131 Naples, Italy

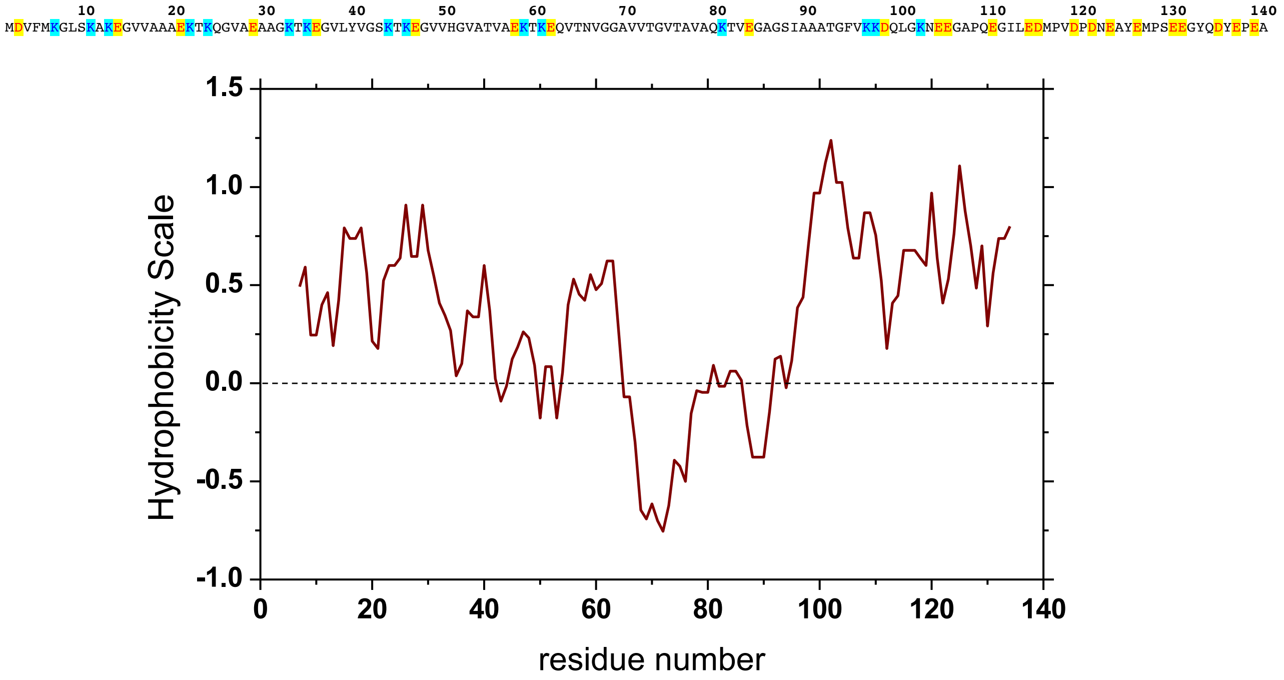

Figure S1. Sequence properties of αS. The sequence of αS is reported on the top of the figure, by highlighting in yellow and blue the positively and negatively charged residues, respectively. The sequence shows how the C-terminal region is highly negatively charged, whereas the membrane-binding region (residues 1 – 98) has high abundance of Lys residues. From the sequence, a plot of the hydrophobicity of the protein was calculated using a moving window of 7 residues and the hydrophobicity scale of Hopp and Woods (Hopp and Woods, 1981). The profile identifies the hydrophobic NAC region, whereas N- and C- terminus show hydrophilic profiles.

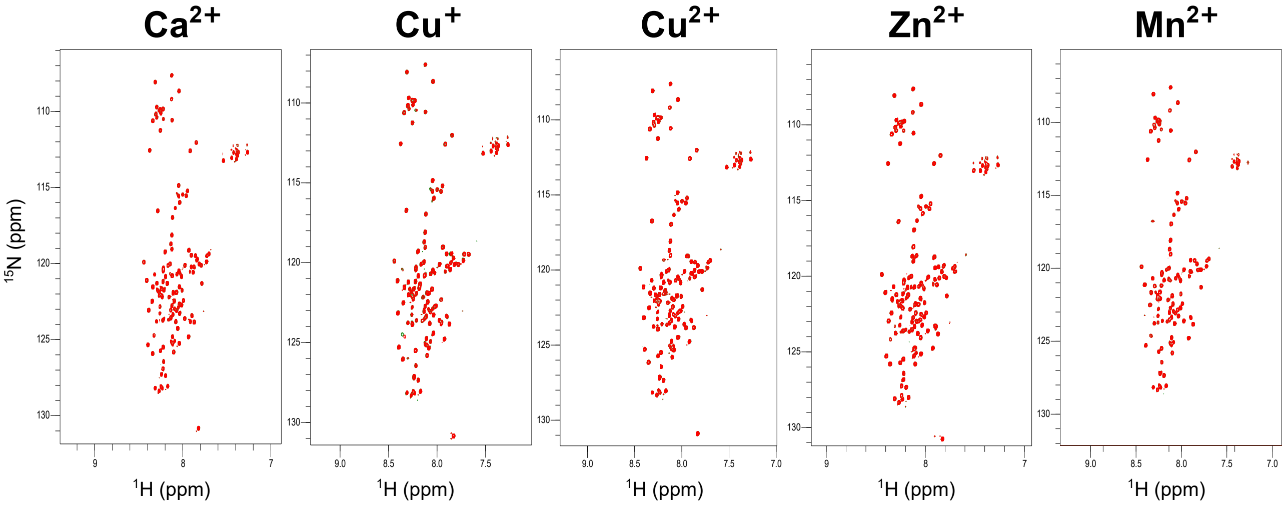

Figure S2. Absence of monomer depletion during CLEANEX measurements. In order to check possible monomer depletion in αS during the measurement of CLEANEX data (10 °C in 25 mM Tris buffer and a pH of 7.0) ^{1}H-^{15}N-HSQC spectra were measured before (green) and after (red) the measurements. The overlap of the spectra indicates no significant peak change due to possible αS monomer depletion in all the metal interactions probed here.

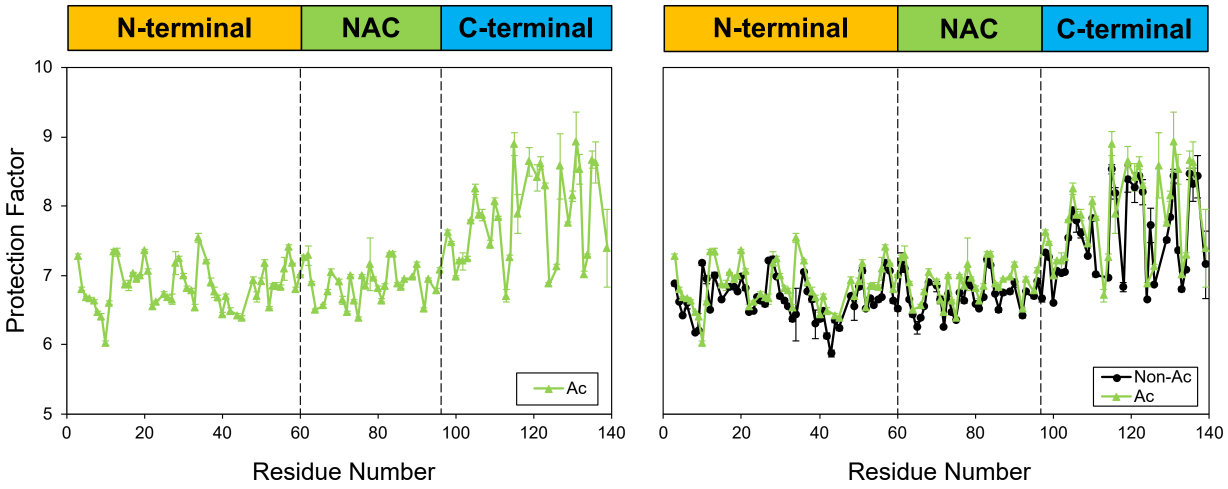

Figure S3. Residue-specific backbone protection factors measured for αS using solution NMR. LogP values from the analysis of CLEANEX-PM data of αS (415 μM) at 10 °C in 25 mM Tris buffer and a pH of 7.0. CLEANEX data were measured using a Bruker spectrometer operating at ^{1}H frequencies of 800 MHz equipped with triple resonance HCN cryo-probe. Left panel shows data measured for acetylated αS, which is the construct employed throughout this work. Right panel overlaps the LogP values measured for non-acetylated and acetylated αS, showing consisting profiles.

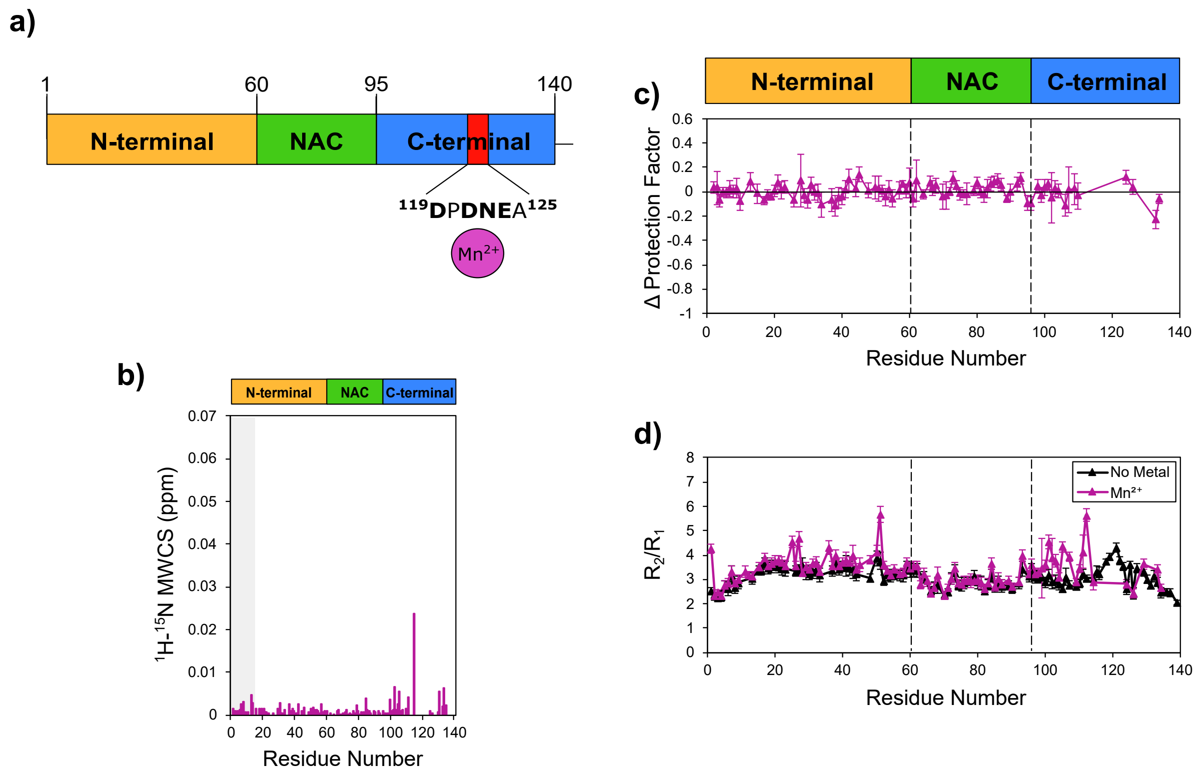

Figure S4. NMR analysis of Mn^{2+} binding to αS. (a) Schematic depicting Mn^{2+} binding by αS. Red regions in the scheme indicate major CSP along the αS sequence upon metal binding. **(b)** Mean weighted CSP (^{1}H-^{15}N MWCS) of αS as a result of the manganese binding. The grey box denotes the first 15 residues of the protein. **(c)** Alteration in the LogP values of αS as a result of Mn^{2+} binding. These values are calculated for each αS residue as the logP of the metal bound state minus the logP of the isolated protein state. **(d)** R_2/R_1 valued from ^{15}N relaxation data of αS in the presence (purple) and absence (black) of manganese (raw data in Figure S6). Error bars are calculated from the fitting errors in R_1 and R_2 measurements. Dotted lines delineate the different regions (N-terminal, NAC and C-terminal) along the sequence of αS. Experiments were performed at 10 °C in 25 mM Tris buffer and a pH of 7.0, and using concentrations of αS and Mn^{2+} of 415 µM and 55 µM, respectively.

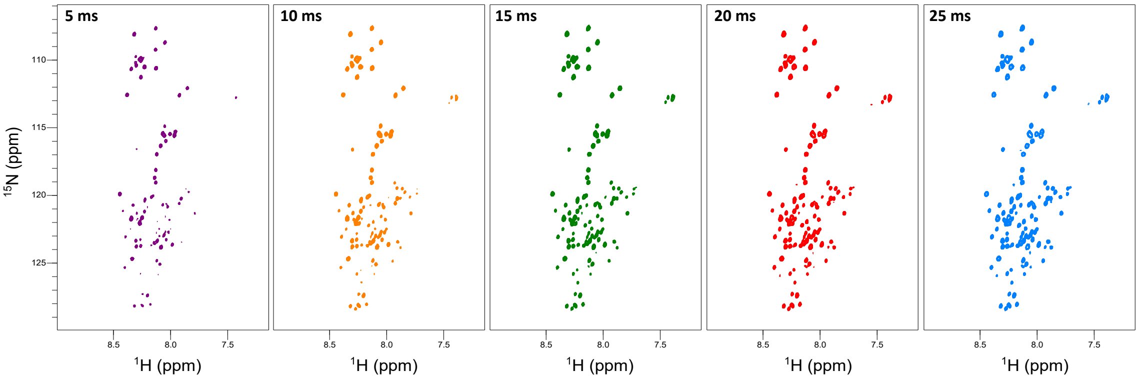

Figure S5. ^{1}H-^{15}N CLEANEX spectra of αS measured using different mixing times. CLEANEX spectra were measured at 10 °C in 25 mM Tris buffer and a pH of 7.0, using a Bruker spectrometer operating at ^{1}H frequencies of 800 MHz equipped with triple resonance HCN cryo-probe.

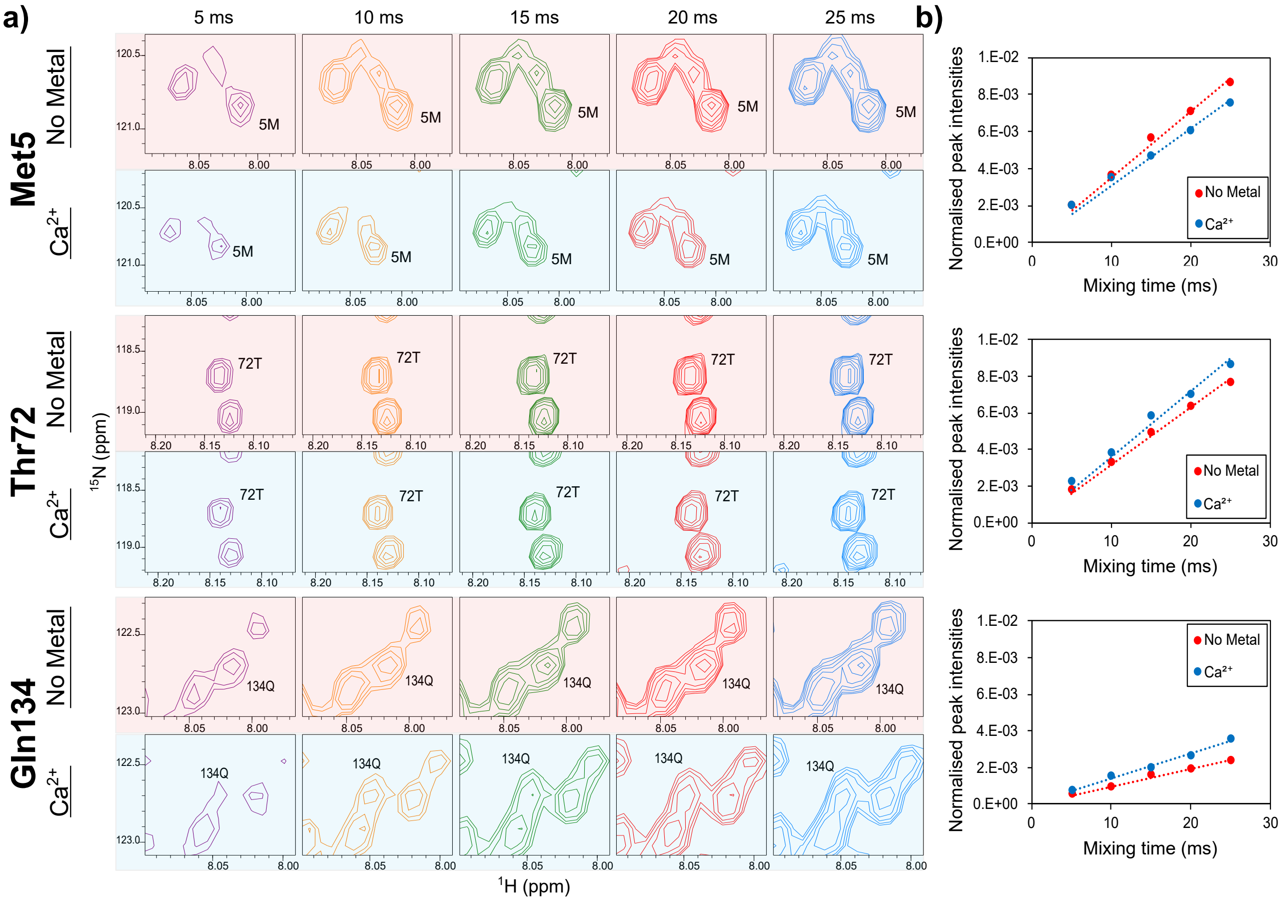

Figure S6. ^{1}H-^{15}N CLEANEX spectra of selected residues of αS. Example of residues M5, T72 and Q134. The data are shown for isolated protein (pink background) and in the presence of calcium (cyan background). Spectra at increasing CLEANEX mixing time are shown (5ms, 10ms, 15ms, 20ms, 25ms). Right panels show fitting of the peak intensities as a function of the mixing times. CLEANEX spectra were measured at 10 °C in 25 mM Tris buffer and a pH of 7.0, using a Bruker spectrometer operating at ^{1}H frequencies of 800 MHz equipped with triple resonance HCN cryo-probe.

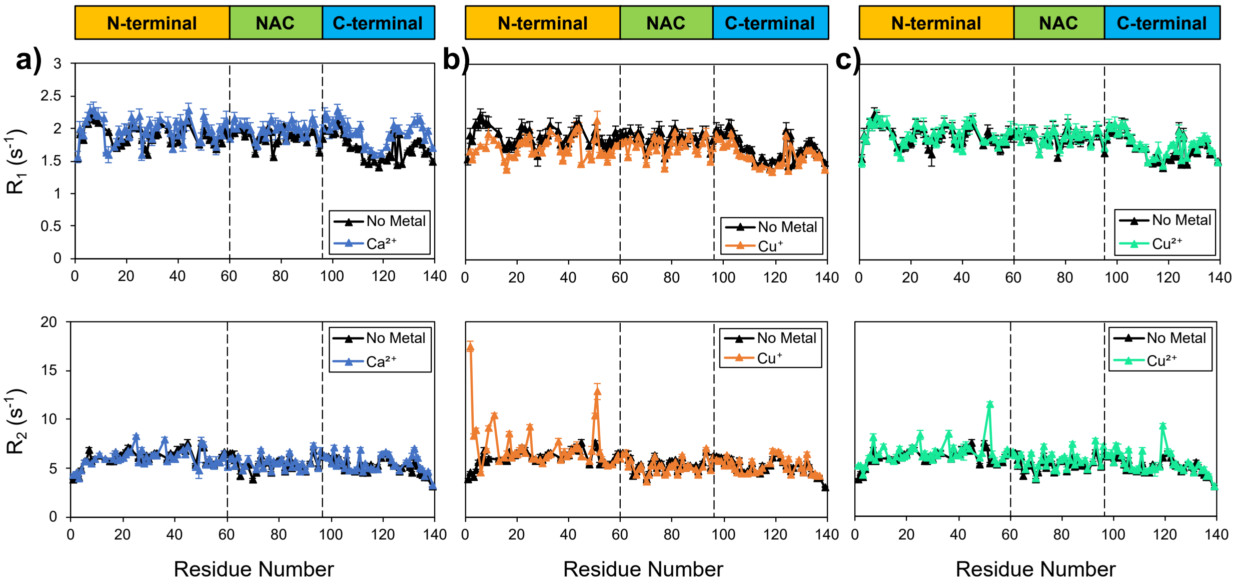

Figure S7. ^{15}N Relaxation data of αS in the presence of calcium and copper. (a) R_1 and R_2 ^{15}N relaxation data of αS in the presence (blue) and absence (black) of calcium. (b) R_1 and R_2 ^{15}N relaxation data of αS in the presence (orange) and absence (black) of Cu^+. (c) R_1 and R_2 ^{15}N relaxation data of αS in the presence (green) and absence (black) of Cu^{2+}. The data were measured at 10 °C in 25 mM Tris buffer and a pH of 7.0, using a Bruker spectrometer operating at ^{1}H frequencies of 800 MHz equipped with triple resonance HCN cryo-probe.

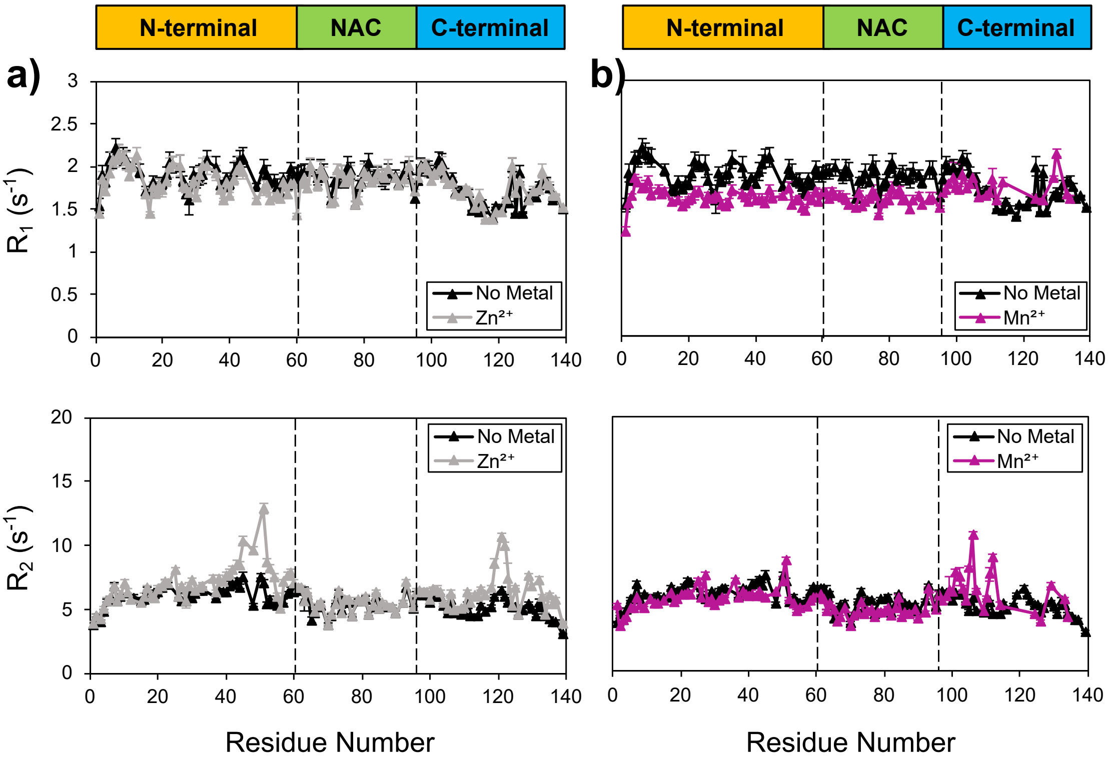

Figure S8. ^{15}N Relaxation data of αS in the presence of zinc and manganese. (a) R_1 and R_2 ^{15}N relaxation data of αS in the presence (grey) and absence (black) of zinc. (b) R_1 and R_2 ^{15}N relaxation data of αS in the presence (purple) and absence (black) of manganese. The data were measured at 10 °C in 25 mM Tris buffer and a pH of 7.0, using a Bruker spectrometer operating at ^{1}H frequencies of 800 MHz equipped with triple resonance HCN cryo-probe.

References

Hopp TP, Woods KR (1981) Prediction of protein antigenic determinants from amino acid sequences. Proc Natl Acad Sci U S A 78, 3824-8, doi:10.1073/pnas.78.6.3824
